# Laser-Treated Screen-Printed Carbon Electrodes for Electrochemiluminescence imaging

**DOI:** 10.1021/cbmi.4c00070

**Published:** 2024-11-22

**Authors:** Claudio Ignazio Santo, Guillermo Conejo-Cuevas, Francesco Paolucci, Francisco Javier Del Campo, Giovanni Valenti

**Affiliations:** † Department of Chemistry “G.Ciamician”, University of Bologna, UE4, Via. P. Gobetti 85, 40129 Bologna, Italy; ‡ BCMaterials, Basque Center for Materials, Applications and Nanostructures, UPV/EHU Science Park, 48940 Leioa, Vizcaya Spain; § IKERBASQUE, Basque Foundation for Science, 48009 Bilbao, Spain

**Keywords:** electrochemiluminescence, screen-printed carbon electrode, laser treatment, beads-based biosensor, ECL microscopy, antibody detection

## Abstract

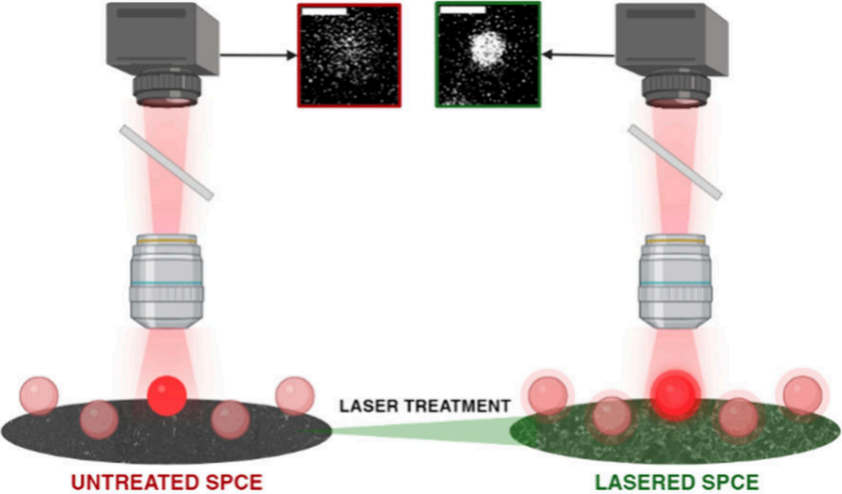

Electrochemiluminescence (ECL) is nowadays a powerful technique widely used in biosensing and imaging, offering high sensitivity and specificity for detecting and mapping biomolecules. Screen-printed electrodes (SPEs) offer a versatile and cost-effective platform for ECL applications due to their ease of fabrication, disposability, and suitability for large-scale production. This research introduces a novel method for improving the ECL characteristics of screen-printed carbon electrodes (SPCEs) through the application of CO_2_ laser treatment following fabrication. Using advanced ECL microscopy, we analyze three distinct carbon paste-based electrodes and show that low-energy laser exposure (ranging from 7 to 12 mJ·cm^–2^) enhances the electrochemical performance of the electrodes. This enhancement results from the selective removal of surface binders and contaminants achieved by the laser treatment. We employed ECL microscopy to characterize the ECL emission using a bead-based system incorporating magnetic microbeads, like those used in commercial platforms. This approach enabled high-resolution spatial mapping of the electrode surface, offering valuable insights into its electrochemical performance. Through quantitative assessment using a photomultiplier tube (PMT), it was observed that GST electrodes could detect biomarkers with high sensitivity, achieving an approximate detection limit (LOD) of 11 antibodies per μm^2^. These findings emphasize the potential of laser-modified GST electrodes in enabling highly sensitive electrochemiluminescent immunoassays and various biosensing applications.

## 1 Introduction

Electrochemiluminescence is a sensitive and versatile methodology, recognized for its numerous benefits, which have established it as a valuable tool across various analytical applications, especially in the field of biosensing.^1,2^

ECL merges the advantages of electrochemical and luminescent methods, providing excellent sensitivity and selectivity, straightforward spatial and temporal control, and a simplified optical arrangement. The generation of light triggered by an electrochemical excitation brings to the ECL a superior signal-to-noise ratio than the common photochemical methods. The development of the *ECL coreactant mechanism* promoted by Bard and co-workers^3^ opened a new field of ECL applications and in recent years to the ECL imaging. This new route of ECL generation involves the use of a coreactant; an auxiliary reagent that, upon electrochemical reaction at the electrode, assists in populating the luminophore’s excited state.^4^ Moreover, this new mechanism finds a perfect application in aqueous media, never performed before for the problem of a restricted water potential window to produce ECL through the annihilation pathway. Since that discovery, ECL has been widely utilized for detecting various substances, from biological molecules to clinical indicators, due to its high sensitivity, low background noise, and controllability.^5^

Today, [Ru(bpy)_3_]^2+^ and Tripropylamine (TPrA), are the most widely used luminophore and coreactant, respectively, in commercially available ECL biosensors. They operate through a *heterogeneous ECL coreactant mechanism*, where the luminophore is positioned away from the electrode surface to prevent direct electron transfer. This configuration is limited by the diffusion of coreactant radicals generated at the electrode.^6,7^ This is commonly achieved by immobilizing the luminophore-tagged biorecognition component onto microspheres or microplate wells. Such a strategy is employed in systems like Elecsys and Meso Scale Discovery analyzers.^2,8,9^ ECL immunoassays offer a powerful platform for the accurate and sensitive detection of biological indicators in diagnostic applications.

The ECL signal is generated via a *low-potential mechanism*, extensively described previously,^3,4^ wherein TPrA is directly oxidized at the electrode surface (1–2). A fraction of the oxidized TPrA is deprotonated (3), forming a stable radical intermediate. This radical species reduces the [Ru(bpy)_3_]^2+^ luminophore to its excited state, [Ru(bpy)_3_]^+^ (4). Concurrently, the continuous oxidation of TPrA at the electrode generates oxidized coreactant species, which can react with [Ru(bpy)_3_]^+^ to form the excited state, [Ru(bpy)_3_]^2+*^ (5). The subsequent relaxation of [Ru(bpy)_3_]^2+*^ to its ground state emits ECL radiation (6).
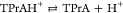
1
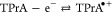
2
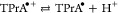
3
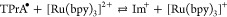
4
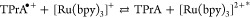
5
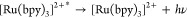
6where Im^+^ (iminium ion) results from the oxidation of TPrA^•^.

The integration of electrochemiluminescence with microscopy (ECLM) has significantly enhanced the optimization of this technique, particularly by enabling the visualization of the physicochemical properties of the electrode surface.^10−13^ This method, known as ECL microscopy, improves the spatial resolution of ECL, facilitating the characterization of electrode surfaces, visualization of individual objects, cells and assessment of their ECL activity.^14,15^

Enhancing the ECL mechanism’s efficiency to optimize its generation is a major goal of ECL research. An essential component in the creation of ECL is electrode material. For effective ECL signal production, optimal electrical conductivity and appropriate optical properties must be ensured by high-quality electrode materials.^16^

Noble metals such as gold and platinum provide optimal conductivity and are well-suited for ECL production, remaining prevalent in many commercial applications. However, their high cost and relatively narrow potential windows restrict their use to specific fields, and they require multiple treatments to maintain good reproducibility. In this context, carbon-based electrode materials offer an ideal compromise between excellent electrochemical properties and affordability. Moreover, incorporating carbon micro- and nanomaterials significantly enhances the electrochemical properties of the electrode. This synergy leverages the inherent advantages of carbon with the benefits of nanoscale dimensions, such as a large surface area, excellent electrical conductivity, low cost and abundance, chemical and mechanical stability, and ease of fabrication.^17−23^

Carbon-based materials like graphite, graphene, and nanotubes can be readily transformed into pastes and inks, enabling cost-effective, easy-to-fabricate small and disposable electrodes. Screen-printed carbon-based electrodes present high surface areas, excellent conductivity, and durability against chemical and mechanical stress, which are fundamental properties of disposable electrodes. With a wide potential window suitable for diverse electrochemical applications, carbon-based electrodes are versatile tools in sensors, biosensors, and beyond.^24,25^

However, the production of screen-printed electrodes also meets challenges, largely due to the use of so-called binders. Binders are (electrochemically) inert polymeric or elastomeric materials that are essential for ensuring the optimal dispersion of carbon particles, managing the viscosity of the solution, guaranteeing good adherence to the surface, and achieving the desired print thickness of the final structures. However, they often have additional effects on the final functionality of printed structures. For instance, too high binder concentrations can result in structures of lower conductivity, or undesirable surface properties such as superhydrophobicity and poor electron transfer rates.^26,27^

Typical binders used in commercial inks may include various cellulosic materials, such as ethyl cellulose and nitrocellulose,^28^ acrylates, such as methyl-methacrylate-based,^29^ and even fluorinated polymers, such as Viton.^30^ The choice of a specific binder depends on the application, which implies adhesion to a particular kind of substrate, particular chemical and thermal resistance and compatibility, and resistance to wear, among other considerations.

Some commercial graphite inks are designed for the construction of electrodes suitable for electrochemical applications, such as the case of Gwent and Henkel pastes used here. Other inks, such as the GST ink also used in this work, may be designed as multipurpose, affordable conducting inks to replace more expensive metal-based inks in a wide range of applications beyond electrochemistry. All these inks are thermally cured at a moderate temperature, to evaporate the solvent without damaging the binder composition, which ensures good print adhesion and mechanical stability. Under these circumstances, it is not possible to obtain a graphite-only surface, and binder residues are always present. These binder residues are detrimental to electrochemical processes, which has motivated the investigation of surface cleaning and activation protocols.^31^ Most of these methods rely on wet methods, which limit their practical use. Laser treatment, in contrast, can be applied as part of the fabrication process, so that the user does not need to apply any preconditioning steps.

Here, we propose the use of carbon-based screen-printed electrodes treated with laser postprocessing to enhance their electrochemical properties, ensuring improved ECL performance. Employing a CO_2_ laser at low energy densities (7–12 mJ·cm^–2^) under atmospheric conditions removes surface impurities, including binders, and induces the formation of more crystalline graphite with larger surface areas. This results in improved electron transfer and altered wettability, which collectively impact ECL generation.^32,33^

## 2 Experimental Section

All the materials were purchased from Sigma-Aldrich. 2.8 μm beads coated with streptavidin were obtained from ThermoFisher Scientific (Dynabeads beads).

### 2.1 Electrode Fabrication and Characterization

Graphite electrodes were screen-printed following previously reported methods.^33,34^ Briefly, Autostat WP20 substrates (MacDermid Autotype, UK) were screen-printed with carbon pastes C20305194P4 and GST 4500 (Sun Chemical-Servilan, ES) and Loctite EDAG 440B (Tetrachim, FR). These electrodes will be referred to as Gwent, GST, and Henkel, respectively. Silver paste Loctite EDAG 725A and UV-curable dielectric Loctite EDAG PF 455B (Tetrachim, FR) were also used. Electrode designs were created using VectorWorks 2024 Student Edition (Techlimits, ES). Film-positives and screens with 77 threads·cm^–1^ and 40 μm fiber diameter SEFAR 1500 fabric were fabricated on aluminum frames (Paymser, ES).

Silver contacts and tracks, and pseudoreference electrodes were first printed on WP20 Polyethylene terephthalate (PET) substrates and cured in a convection oven at 115 °C for 15 min. Next, graphite working and auxiliary electrodes were printed using the inks described above, and cured as per their respective technical datasheets. To protect the conductive tracks and delineate the working, auxiliary, and pseudoreference electrode regions, a dielectric coating was applied. This dielectric coating was cured by flood exposure in a UVAcube 400 UV lamp (Honle UV Technology, DE) for 45 s. Laser postprocessing was carried out using a 30W Epilog Mini 18 engraver (Epilog, US) under raster mode at 600 dpi. Raster speed was fixed at 50%, equivalent to approximately 830–860 mm·s^–1^. Laser power was fixed between 9 and 12%, with corresponding applied energies between 7.7 and 10.2 mJ·cm^–2^. Subsequent to treatment, the electrodes were vacuum-sealed in RP-1 Agent (Mitsubishi Gas Company, JP) gas-barrier bags to preserve their integrity. The morphology of the working electrodes was analyzed using scanning electron microscopy (FEG-SEM Hitachi S-4800) at 15 kV. Raman spectroscopy was performed using an InVia Raman spectrometer (Renishaw) equipped with a Leica DMLM microscope and an argon ion laser (Modu-Laser) with a 514 nm wavelength. X-ray photoelectron spectroscopy (XPS) measurements were conducted using a SPECS system (Berlin, Germany) equipped with a Phoibos 150 1D-DLD detector and a monochromatic Al Kα X-ray source (1486.7 eV). Contact angle analysis was carried out using an Ossila contact angle goniometer (software version 3.1.2.2 for Windows 10). A 2 μL water droplet was used to determine the contact angle in air.

### 2.2 Functionalization of 2.8 μm Beads with Ru(bpy)_2_-bpy-NHS

The 2.8 μm diameter magnetic beads were functionalized with Ru-NHS (ruthenium *N*-hydroxysuccinimide) diluted in 0.01 M PBS buffer (1×, pH = 7.4) to a concentration of 2.7 × 10^–2^ mg·mL^–1^.

For the functionalization, 217 μL of 2.8 μm beads were taken and washed twice in PBS using magnetic support (PureProteome Magnetic Stand, Sigma-Aldrich). After washing, the supernatant was removed, and the Ruthenium solution was added. The solution was continuously stirred until the next day to optimize bead functionalization.

The next day, the beads were washed three times in 1x PBS, removing the supernatant each time. Finally, they were dispersed in 217 μL of PBS to maintain the initial beads’ concentration (0.72 mg·mL^–1^).

### 2.3 Functionalization of Antibody with Biotin and [Ru(bpy)_3_]^2+^

The procedure has been described in our previous work.^35^ The antibody (IgG from Vector Laboratories) was functionalized by incubating a 1 mg·mL^–1^ solution in PBS with a high molar excess of biotin, EDC, NHS, and [Ru(bpy)_2_(mcbpy-O-Su-ester)] (85 equiv). After a 90 min incubation, the solution was purified using Millipore Amicon Ultra 0.5 mL centrifugal filter devices with a 5 kDa cutoff membrane to remove excess ruthenium complex and biotin.

### 2.4 Functionalization of 2.8 μm Beads with Ru-Labeled Biotinylated Antibody

A solution of 1 nM of antibody labeled with biotin and [Ru(bpy)_3_]^2+^ was used for the beads’ functionalization.

800 μL of the bead solution was pipetted into a 20 mL tube, magnetically gathered for 2 min, and the supernatant was removed. The beads were then washed twice with 10 mL of 0.01 M PBS for 5 min each. The beads were then incubated with 18 mL of Ru-labeled antibody solution for 3 h at 37 °C on a tube rotator. The beads were magnetically collected for 2 min, and the supernatant was removed. Finally, beads were stored in PBS with a total volume of 800 μL, to maintain the initial concentration of the beads’ solution. The same procedure was used with a progressive dilution of the starting solution of biotinylated Ru-labeled antibody to obtain beads functionalized with different antibody loadings.

### 2.5 Single-Bead ECL Microscopy Analysis

The ECL/optical imaging was performed using a *Raman ec flow cell attachment for SPE holder* with homemade modifications, comprising SPCE as working electrode (0.096 cm^2^). Pt wire and Ag/AgCl (3 M KCl) were used as counter and reference electrodes, respectively. The direct microscope is from Nikon (Chiyoda, Tokyo, Japan) and can work either in transmission or in reflection mode. The microscope was shielded from external light by a custom-made dark box and was fitted with a motorized stage (Corvus, Marzhauser, Wetzlar, Germany) for electrochemical cell positioning and right focal plane observing.

For image acquisition, an ultrasensitive Electron-Multiplying CCD camera (EM-CCD 9100–13 from Hamamatsu, Hamamatsu Japan) with a resolution of 512 × 512 pixel and a size of 16 × 16 μm was connected to the microscope. Finally, a long-distance water dipping objective was used (100x, 1.1 numerical aperture, 2.5 mm working distance).

The integrated system also includes a potentiostat from BioLogic (SP-300) to generate the ECL emission. The EMCCD camera integration time was set to 200 ms, allowing the acquisition of 5 images per second. The system was triggered by the potentiostat, ensuring that image acquisition by the EMCCD commenced instantaneously with the start of the potential scan. A postprocessing step allows the correlation of the ECL intensity in each image to the exact potential scanned at the time the image was captured.

### 2.6 Quantitative ECL Analysis

A photomultiplier tube (Hamamatsu R928) was positioned at a fixed distance above the 3D-printed electrochemical cell to detect ECL signals. To minimize external light interference, both the cell and the PMT were housed within a dark box. A high-voltage power supply with a trans-impedance amplifier (Hamamatsu C6271) provided a 750 V bias to the PMT, triggered externally by the potentiostat’s DAC module. The amplified PMT output signal was acquired by the potentiostat’s ADC module (BioLogic SP-300) to generate light/current/voltage graphs.

## 3 Results and Discussion

Our initial study focuses on the characterization of SPCE made with three different carbon pastes, which will be named Gwent, Henkel and GST in this work. XPS characterization (Figure S1) shows the removal of sp^3^ carbon, present mainly in the binder polymer chains, and the enrichment or predominance of sp^2^ carbon following laser treatment. Raman spectra also confirm laser treatment increases the G (1600 cm^–1^) band relative to the D (1360 cm^–1^) band, which means more crystalline graphite has emerged (Figure S2). Streptavidin-coated microbeads were functionalized with Ru(bpy)_2_-bpy-NHS (ruthenium *N*-hydroxysuccinimide) (Figure S3), exploiting the NH_2_ sites present on the streptavidin. These beads offer a substantial benefit by providing a greater number of binding sites, which allows for the immobilization of a maximum Ru loading capacity. To thoroughly assess the ECL emission features, we employed ECL microscopy using an EM-CCD camera (Figure S4 and Supplementary Movie 1). This technique enabled the examination of ECL signals generated by the microbeads, effectively suppressing background noise. This approach allowed for optimal spatial characterization of the electrode surface, including its homogeneity and electrical activity. To mimic a commercial ECL immunoassay system, the ECL from microbeads targeted with a Ru-labeled biotinylated antibody (Figure S5) was quantitatively measured using a photomultiplier tube detector.^34^

**Figure 1:**
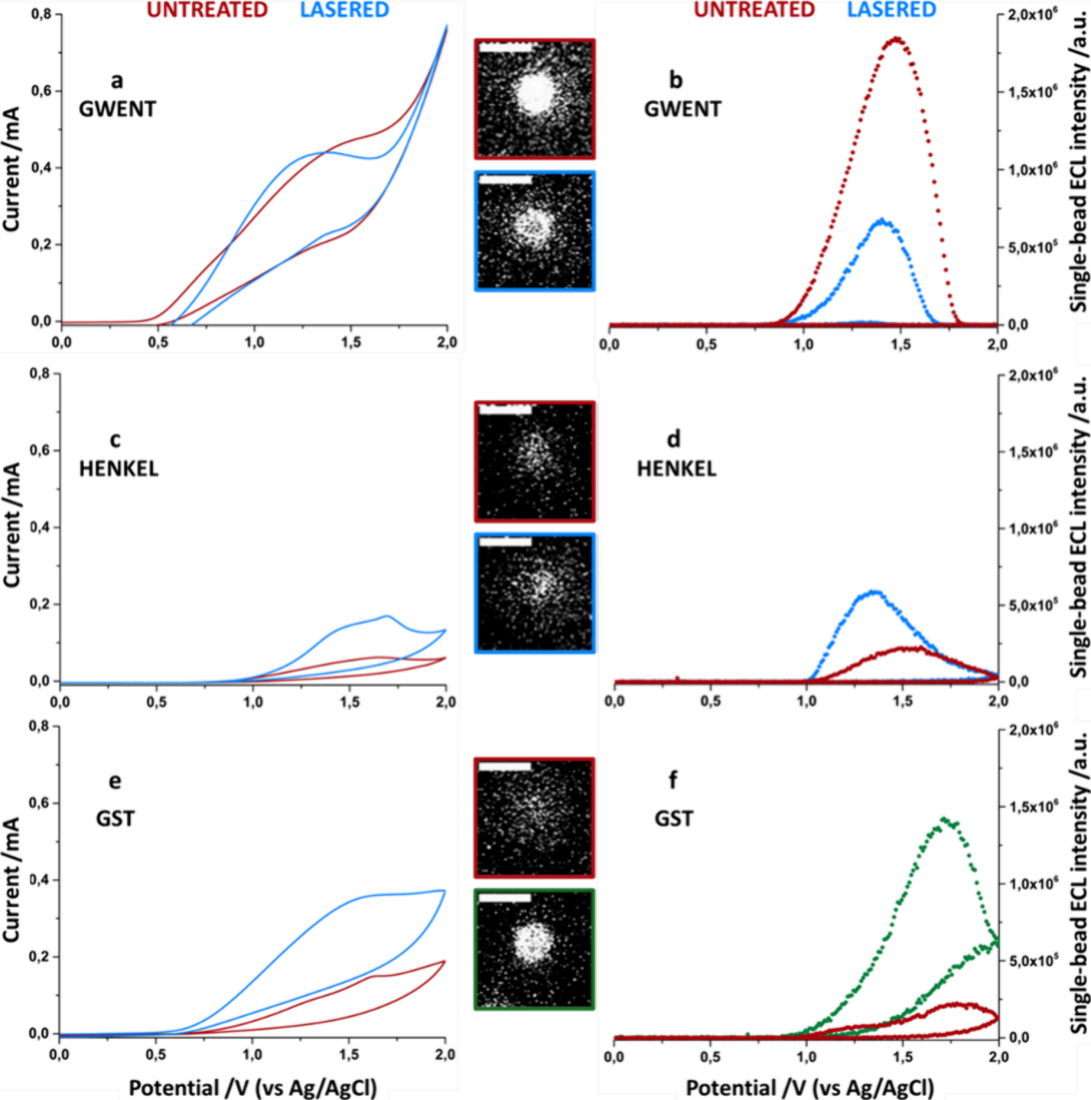
Single-bead ECL intensity collected during the cyclic voltammetry for Gwent, Henkel and GST electrodes before and after the laser treatment. In graphs **a**, **c** and **e**, the current trend for the three electrodes before and after laser treatment, red and blue lines respectively, are shown. Dotted lines, graphs **b**, **d** and **f**, represent the single-bead ECL intensity at each 200 ms. Green dotted lines correspond to the ECL intensity of the best lasered electrode. The inset images show the ECL emission of the individual beads taken at the maximum of the cyclic voltammetry ECL emission (Scale bar 5 μm). Potential scan from 0 V vs OCP to 2 V vs ref, ref (reference electrode) is Ag/AgCl (KCl sat.), the counter electrode is Pt wire, scan rate 50 mV·s^–1^, EM-CCD integration time 200 ms, sensitivity gain 800, gain 5, magnification 100x. Image processing involves integrating a 50 × 50 pixel square area centered on the magnetic bead across all analysis frames (one image captured every 200 ms). The background is calculated from the same area on a bead that is not functionalized with ruthenium and subtracted from the signal of the functionalized bead. Finally, each image is correlated with the applied potential at that moment to obtain a trend of the ECL intensity as a function of the scanned potential. Each measurement is an average of at least *N* = 5 magnetic beads.

Single-bead ECLM revealed an increase in current for Henkel and GST electrodes (Figure 1c,e), accompanied by an enhanced single-bead ECL intensity (Figure 1d,f) after laser treatment. In the case of the Gwent electrode, there was no notable alteration in the current (Figure 1a) and a reduction in the ECL signal (Figure 1b). Notably, for Gwent and Henkel SPEs, the pronounced increase in hydrophobicity^33^ has resulted in a lack of reproducibility of measurements and between different beads deposited on the same SPCE.

**Figure 2:**
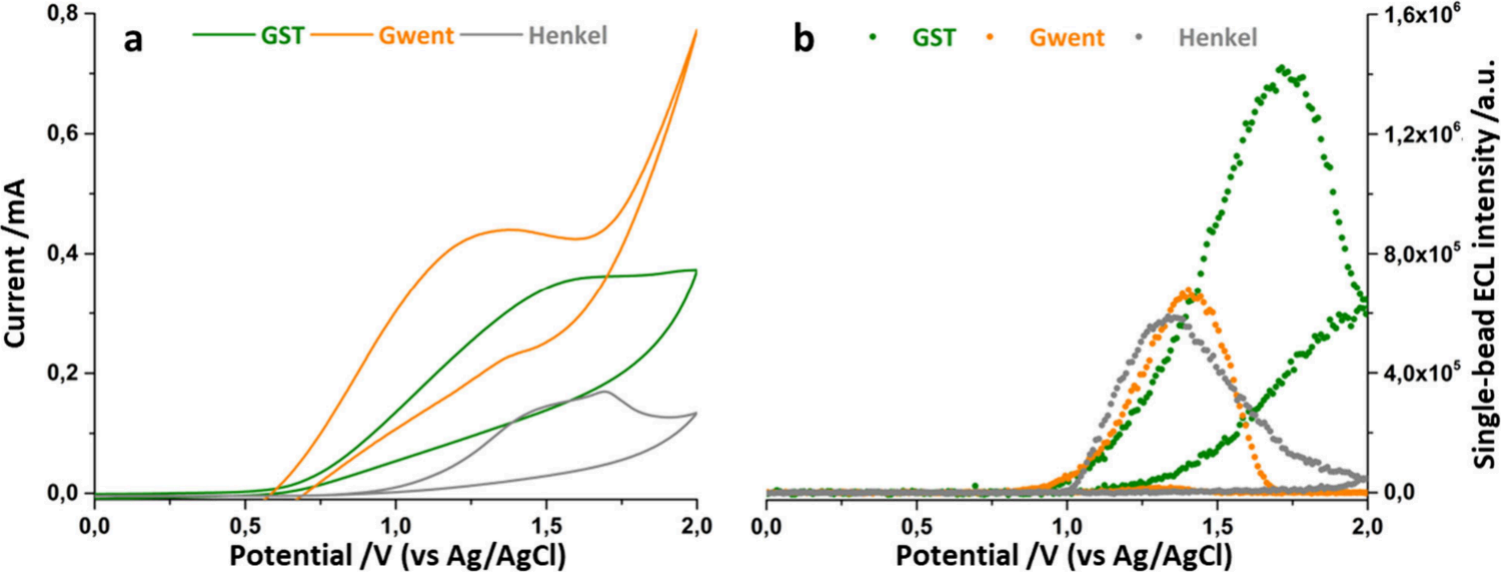
Comparison between single-bead current **a)** and ECL signal **b)** during cyclic voltammetry of laser-treated SPEs produced with different pastes. Scan rate 50 mV·s^–1^, EM-CCD integration time 200 ms, sensitivity gain 800, gain 5, magnification 100×. Each measurement is an average of at least *N* = 5 magnetic beads.

Although laser treatment of graphite electrodes enhances electron transfer rates (Figure 2a), surface wettability may also be impaired depending on the paste composition and morphology. As the material becomes more graphitic, electron transfer is enhanced, but hydrophobicity also increases. This greater hydrophobicity limits solution access to the entire electrode surface, significantly reducing ECL generation for Gwent and Henkel SPEs (Figure 2b and Table 1).

**Table 1:** Alteration of Contact Angles in Three-Electrode Materials Induced by Laser Treatment

electrodes	contact angle before laser	contact angle after laser
Gwent	102°	155°
Henkel	102°	140°
GST	84°	48°

Table 1 summarizes the contact angle analysis of the different electrodes before and after laser treatment. Gwent and Henkel electrodes exhibited a substantial rise in contact angle, from 102° to 155° and 102° to 140°, respectively. This increase in contact angle indicates an growth in surface hydrophobicity. While Gwent electrodes experienced similar reductions in ECL generation after laser treatment, the ECL yielded at laser-treated GST electrodes became the brightest in the series, highlighting the critical role of electrode wettability.^33^

In the case of GST, although the removal of amorphous carbon also exposes more crystalline graphite, which is mainly responsible for the hydrophobic behavior observed, the binder is much more effectively removed than in the case of Henkel and Gwent electrodes, leaving a more porous surface (SEM images in Figures S6–S9). This higher porosity compensates for the hydrophobicity of the graphite and allows the solution to permeate the printed layer, thus improving wetting.

Having established that the GST screen-printed electrode demonstrated superior performance in both ECL emission and reproducibility, subsequent experiments involved consistently depositing a uniform volume of beads onto the electrode and using a photomultiplier tube as a detector (Figure S10). The feasibility of utilizing the SPCE for quantitative analysis to determine different antibody concentrations was evaluated, aiming to establish the electrode as a platform for biomarker detection.

For quantitative bead measurements, a custom cell was fabricated using a Fused Deposition Modeling (FDM) printer (Ultimaker S33D) as shown in Figure S11. In commercial systems, the Ru content on the magnetic microbead surface is proportional to the immobilized analyte. In our setup, biotinylated antibodies functionalized with the Ru dye were immobilized on the beads. Knowing the antibody concentration, the bound concentration of [Ru(bpy)_3_]^2+^ was determined using UV–vis spectroscopy by calculating the [Ru(bpy)_3_]^2+^ concentration from the MLCT absorption band at 450 nm, within an absorbance range below 0.1, based on the Lambert–Beer law. Given that up to 6 Ru complexes can be directly immobilized on an antibody, the minimum detectable amount of antibody can be determined.

Various bead samples were prepared to assess if the selected electrode could provide an ECL response proportional to the antibody amount on the beads. Different antibody quantities were incorporated into the beads using progressively more diluted solutions for bead functionalization, with the most concentrated solution corresponding to a final concentration of 4392 Ru dyes·μm^–2^.

**Figure 3:**
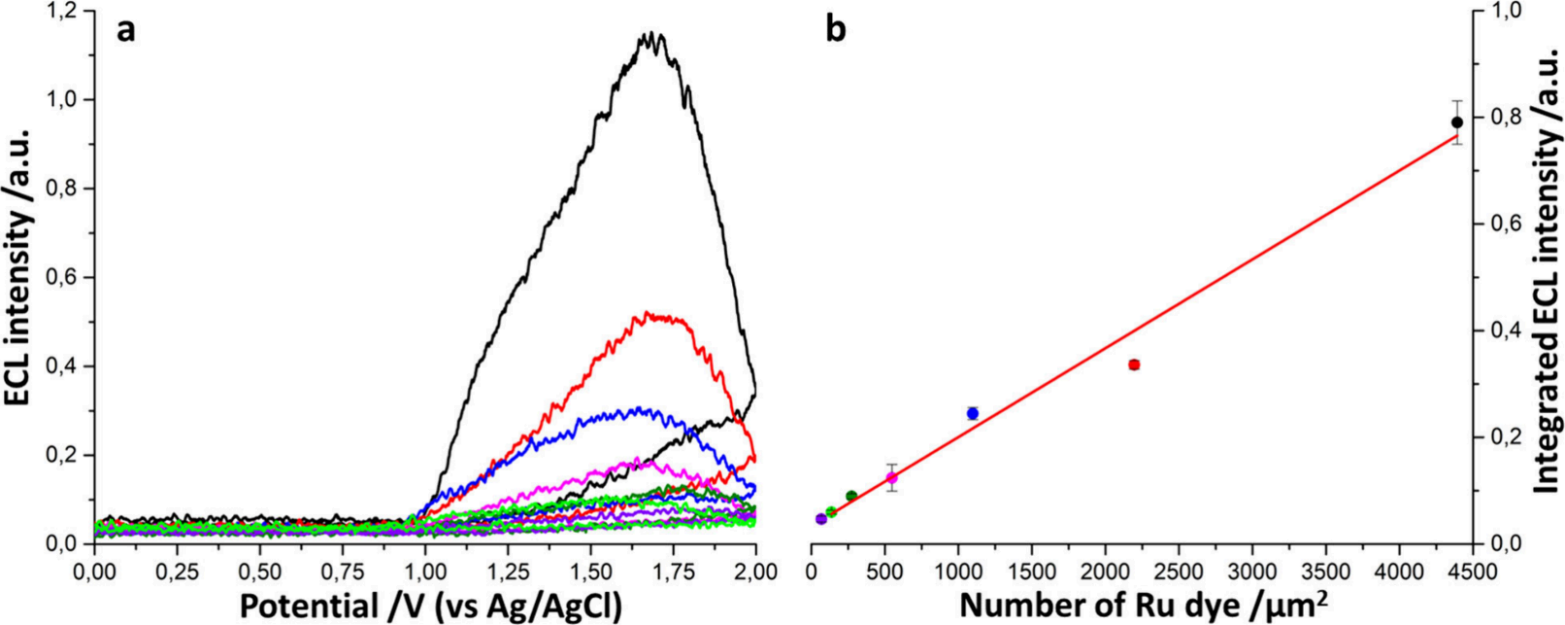
**a)** Quantitative beads ECL intensity during cyclic voltammetry for different [Ru(bpy)_3_]^2+^ concentrations: 4392 (black line); 2196 (red line); 1098 (blue line); 549 (pink line); 275 (dark green line); 137 (light green line); 69 (purple line) Ru dyes·μm^–2^ quantified by previous ICP-MS analysis. **b)** Calibration curve obtained after the integration of the area below the ECL curve plotted as a function of the Ru amount for each bead. Scan rate 50 mV·s^–1^, PMT current amplification 000.0 nA. The equation of the linear calibration curve is *Y* = (1.7 × 10^–4^ ± 1.0 × 10^–5^)*X* + (3.4 × 10^–2^ ± 2.1 × 10^–2^), *R*^2^ = 0.98, where *Y* is the integrated ECL signal and *X* is the number of Ru dye per μm^2^ of bead’s surface. Each datum is the mean ± SD of three independent experiments.

ECL signal decreases with decreasing ruthenium concentration on the beads (Figure 3a). Plotting the integral of the ECL curves against the concentration of ruthenium on the beads yields a calibration curve that interpolates the points quite well without outliers (Figure 3b). Evaluating the blank signal with the same experimental setup with a not-functionalized magnetic microbead, we calculated the limit of detection as the minimum concentration we can detect in our experimental conditions which results in a LOD of 65 Ru dyes·μm^–2^, which means around 11 antibodies·μm^–2^.

## 4 Conclusions

This study investigated the effects of postfabrication laser treatment on screen-printed carbon electrodes made from three different carbon pastes. We found that laser treatment significantly improved the electrochemical and electrochemiluminescence properties of Henkel and GST electrodes, while it did not enhance, and even reduce, the ECL signal for Gwent electrodes. GST electrodes, in particular, showed superior ECL performance and reproducibility.

Using ECL microscopy, we visualized and analyzed single-bead ECL signals, providing detailed insights into electrode surface properties. Quantitative analysis demonstrated the high sensitivity of GST electrodes for biomarker detection, with a limit of detection corresponding to about 11 antibodies·μm^–2^. These results suggest that laser-treated GST electrodes are well-suited for commercial ECL immunoassays and other biosensing applications, offering enhanced sensitivity and reliability. Future work should further optimize laser treatment parameters to enhance electrode performance across different compositions.

## Data Availability

Experimental data are available in AMS Acta at https://amsacta.unibo.it/id/eprint/7883.

## Supporting Information Available

Additional experimental details, the experimental setup of single-bead ECL microscopy and collective beads analysis. characterization of SPCEs surface (XPS, Raman and SEM) (PDF)
Supplementary Movie 1: ECL emission from single beads during cyclic voltammetry on a laser-treated GST SPCE, illustrating the dynamic change in ECL intensity as the applied potential varies (MP4)
