# Supplementary material for: Laser-Treated Screen-Printed Carbon Electrodes for Electrochemiluminescence imaging
Source: Chem Biomed Imaging. 2024 Nov 22;2(12):835–41. doi: 10.1021/cbmi.4c00070 (PMC11672215; doi:10.1021/cbmi.4c00070)
Supplement: Supplementary file 1 — im4c00070_si_001.pdf [file im4c00070_si_001.pdf]

Supporting information

Laser-Treated Screen-Printed Carbon Electrodes for Electrochemiluminescence imaging

Claudio Ignazio Santo,[†] Guillermo Conejo-Cuevas,[‡] Francesco Paolucci,[†] Francisco Javier Del Campo[*,‡,§] and Giovanni Valenti.[*,†]

[†] Department of Chemistry "G.Ciamician", University of Bologna, UE4, Via. P. Gobetti 85, 40129, Bologna, Italy.

[‡] BCMaterials, Basque Center for Materials, Applications and Nanostructures, UPV/EHU Science Park, 48940, Leioa, Vizcaya, Spain.

[§] IKERBASQUE, Basque Foundation for Science, 48009, Bilbao, Spain.

* Email: g.valenti@unibo.it.

* Email: javier.delcampo@bcmaterials.net.

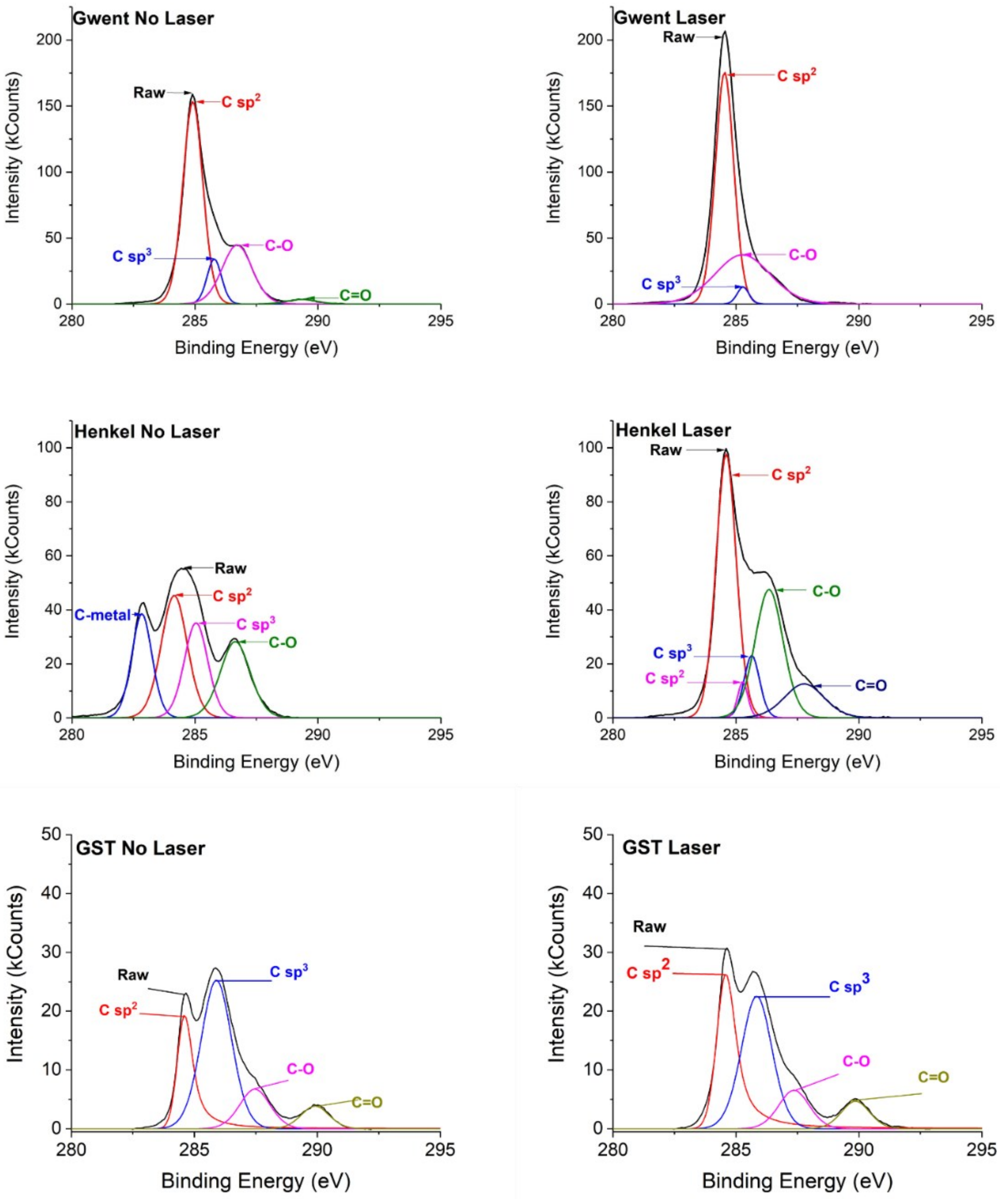

Figure S1. XPS spectra of the different electrodes, before and after laser treatment. Spectra for Henkel and Gwent electrodes are reproduced with permission from Elsevier [1]. XPS measurements were performed with a SPECS system (Berlin, Germany) equipped with Phoibos 150 1D-DLD and monochromatic irradiation source Al Kα (1486.7 eV).

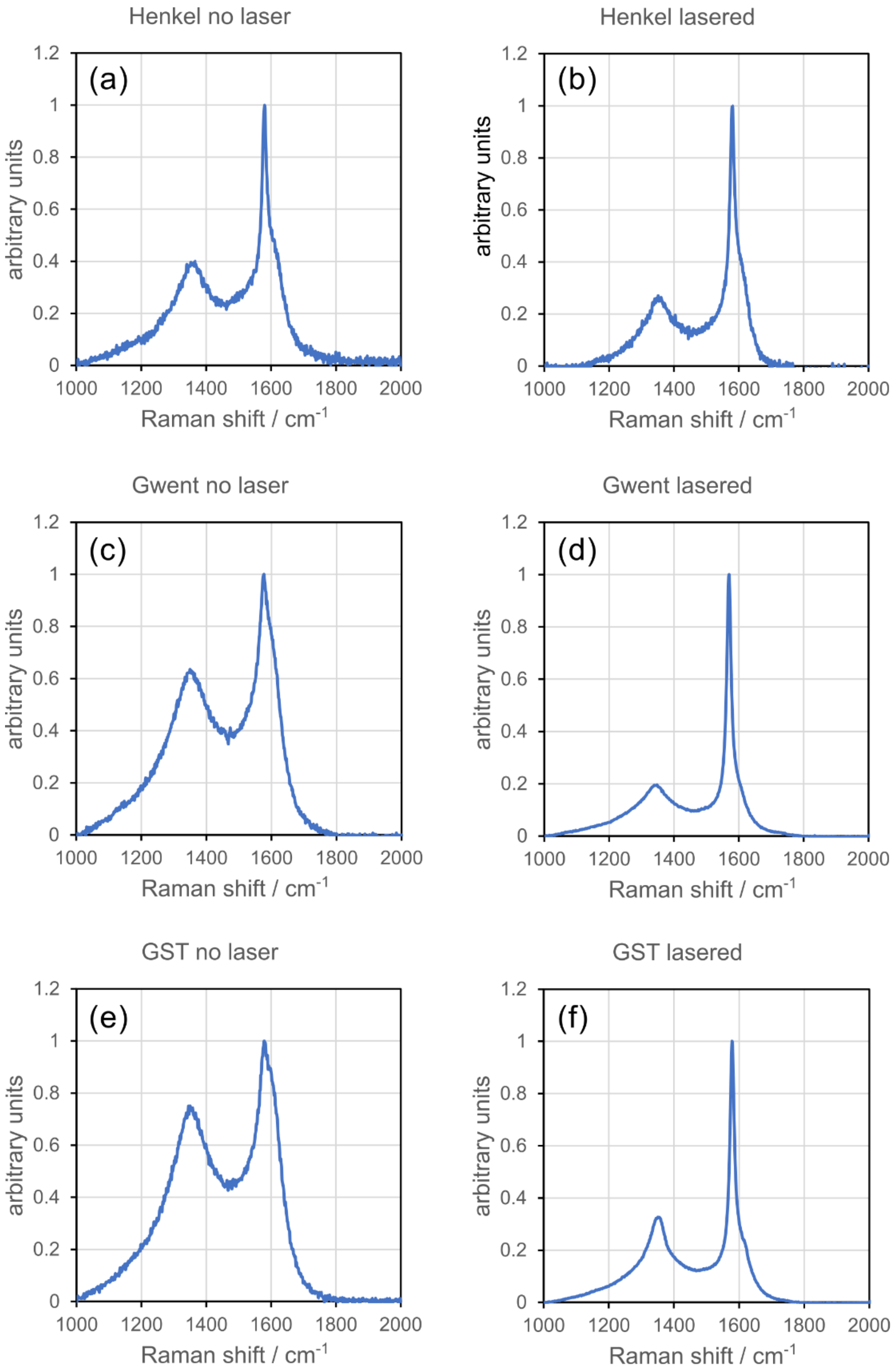

Figure S2. Raman spectra corresponding to the different screen-printed graphite electrodes before (as printed, **a**, **c**, **e**) and after (**b**, **d**, **f**) laser treatment. Raman measurements were carried out using an InVia Raman of Renishaw equipped with a microscope Leica DMLM. A Modu-Laser brand argon ion laser with a wavelength of 514 nm was used.

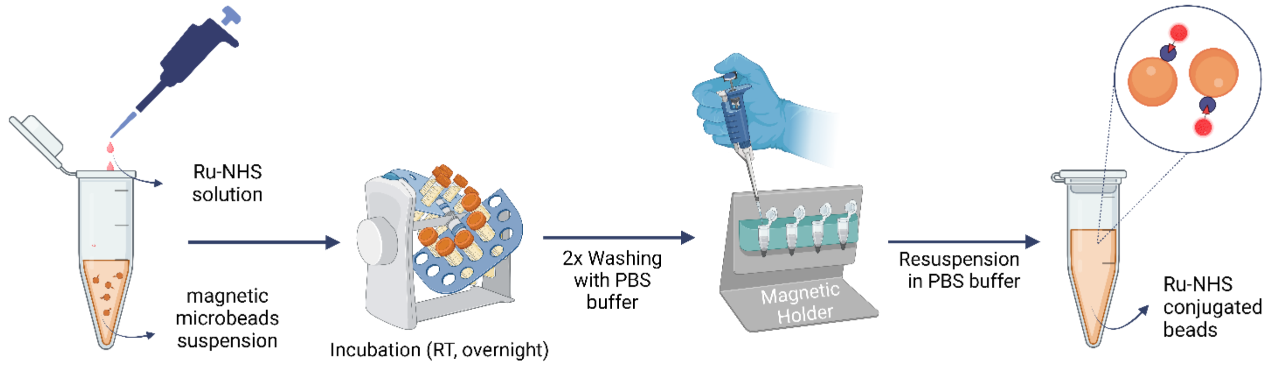

Figure S3. Schematic representation of beads functionalization with Ru(bpy)$_2$-bpy-NHS (Ru(bpy)$_2$(mcbpy-O-Su-ester) (Ru@beads). The 2.8 µm diameter magnetic beads were functionalized with Ru-NHS (Ruthenium N-hydroxysuccinimide) diluted in 0.01 M PBS buffer (1x, pH=7.4) to a concentration of 2.7x10^{-2} mg·mL^{-1}. For the functionalization, 217 µL of 2.8 µm beads were taken and washed twice in PBS using magnetic support (PureProteome™ Magnetic Stand, Sigma Aldrich). After washing, the supernatant was removed, and the Ruthenium solution was added. The solution was continuously stirred until the next day to optimize bead functionalization. The next day, the beads were washed three times in 1x PBS, removing the supernatant each time. Finally, they were dispersed in 217 µL of PBS to maintain the initial beads' concentration (0.72 mg·mL^{-1}).

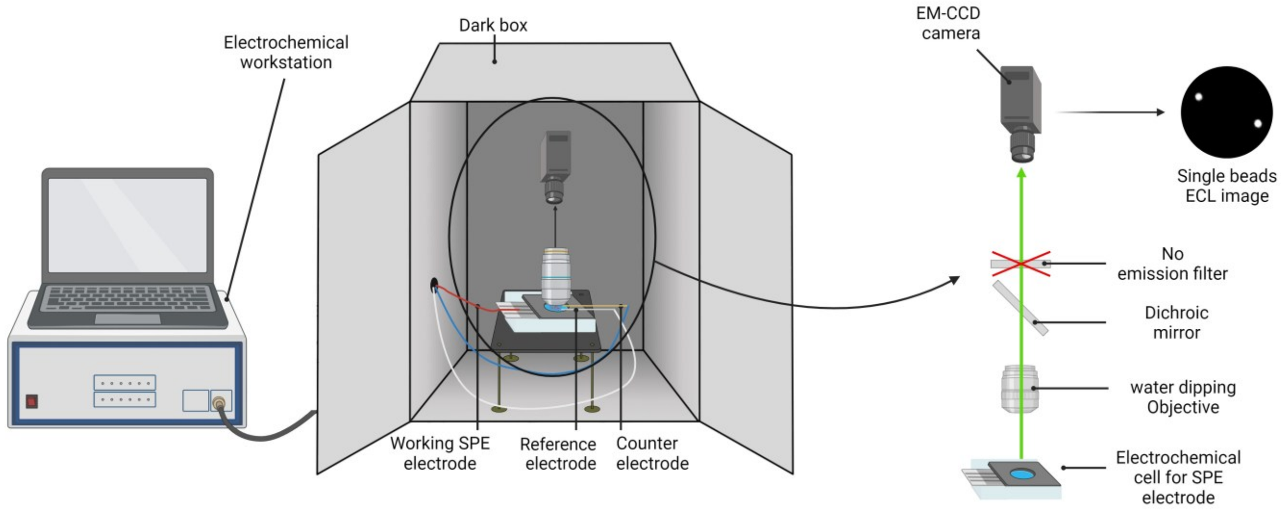

Figure S4. Schematic representation of single beads ECL experimental set-up. The ECL/optical imaging was performed using a *Raman ec flow cell attachment for SPE holder* with homemade modifications, comprising SPCE as working electrode (0.096 cm^2). Pt wire and Ag/AgCl (3 M KCl) were used as counter and reference electrodes, respectively. The direct microscope is from Nikon (Chiyoda, Tokyo, Japan) and can work either in transmission or in reflection mode. The microscope was shielded from external light by a custom-made dark box and was fitted with a motorized stage (Corvus, Marzhauser, Wetzlar, Germany) for electrochemical cell positioning and right focal plane observing. For image acquisition, an ultrasensitive Electron-Multiplying CCD camera (EM-CCD 9100-13 from Hamamatsu, Hamamatsu Japan) with a resolution of 512x512 pixel with a size of 16x16 µm. Finally, a long-distance water dipping objective was used (100x, 1.1 numerical aperture, 2.5 mm working distance). The integrated system also includes a potentiostat from BioLogic to generate the ECL emission. The EMCCD camera integration time was set to 200 ms, allowing the

acquisition of 5 images per second. The system was triggered by the potentiostat, ensuring that image acquisition by the EMCCD commenced instantaneously with the start of the potential scan. A post-processing step allows the correlation of the ECL intensity in each image to the exact potential scanned at the time the image was captured.

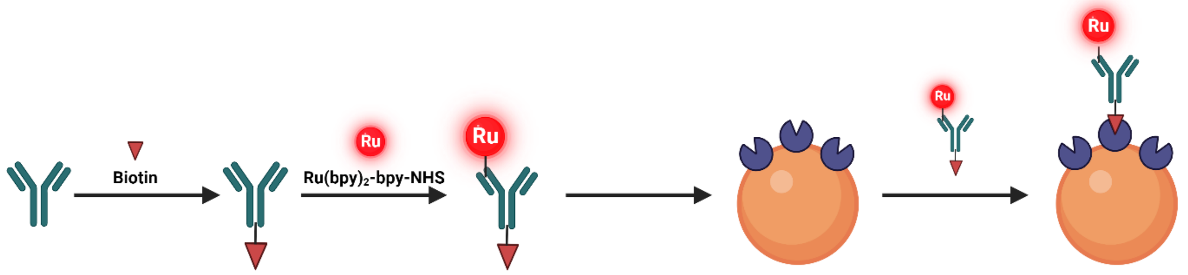

Figure S5. Schematic representation of beads functionalization with $Ru(bpy)_3^{2+}$-labelled biotinylated antibody. The antibody (IgG from Vector Laboratories) was functionalized by incubating a 1 mg·mL^{-1} solution in PBS with a high molar excess of biotin, EDC, NHS, and [$Ru(bpy)_2$(mcbpy-O-Su-ester)] (85 equiv.). After a 90-minute incubation, the solution was purified using Millipore Amicon Ultra 0.5 mL centrifugal filter devices with a 5 kDa cutoff membrane to remove excess ruthenium complex and biotin. A solution of 1 nM of antibody labeled with biotin and $[Ru(bpy)_3]^{2+}$ was used for the beads' functionalization. 800 µL of the bead solution was pipetted into a 20 mL tube, magnetically gathered for 2 minutes, and the supernatant was removed. The beads were then washed twice with 10 mL of 0.01 M PBS for 5 minutes each. The beads were then incubated with 18 mL of Ru-labeled antibody solution for 3 hours at 37°C on a tube rotator. The beads were magnetically collected for 2 minutes, and the supernatant was removed. Finally, beads were stored in PBS with a total volume of 800 µL, to maintain the initial concentration of the beads' solution. The same procedure was used with a progressive dilution of the starting solution of biotinylated Ru-labelled antibody to obtain beads functionalized with different antibody loadings.

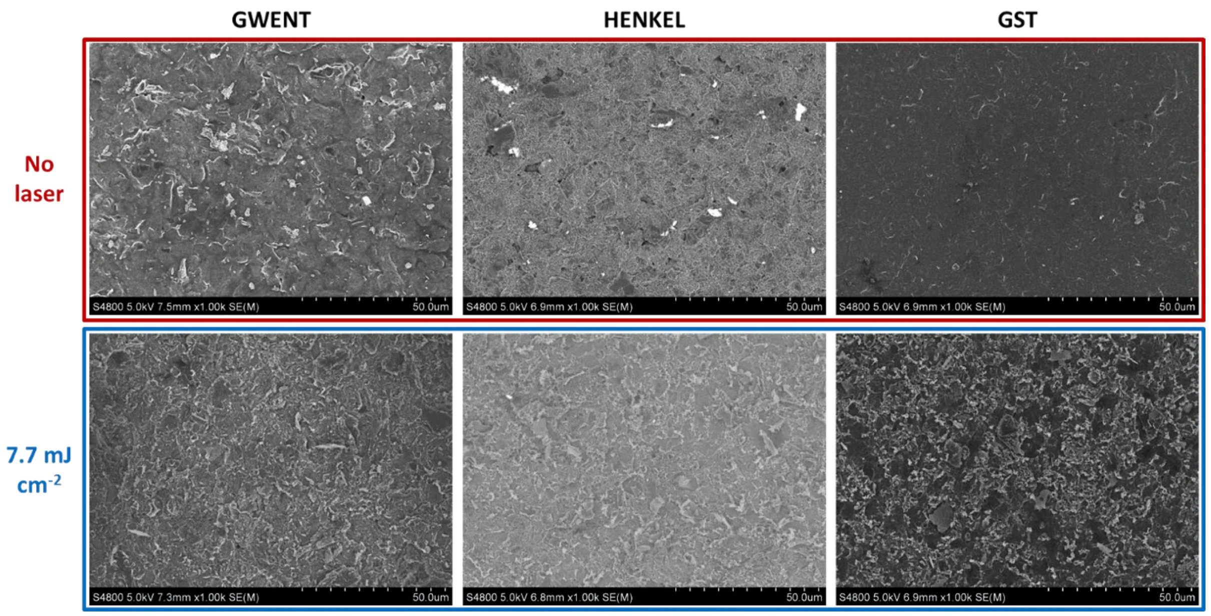

Figure S6. SEM images from the Gwent, Henkel and GST electrodes before and after laser treatment. The morphology of the working electrodes was assessed by scanning electron microscopy (FEG-SEM Hitachi S-4800) at 15 kV.

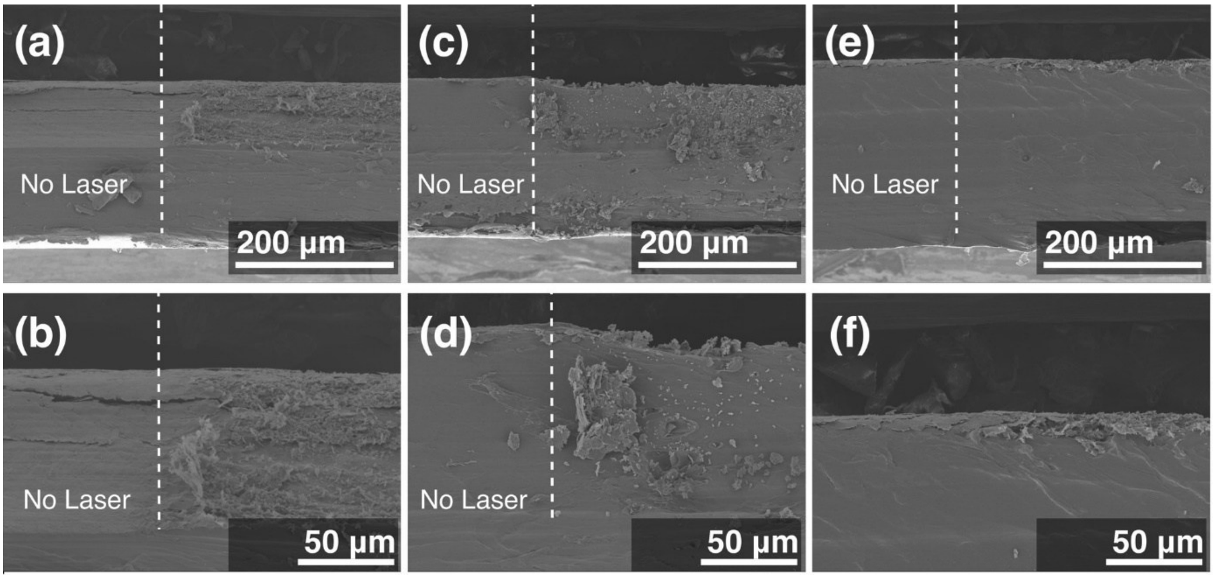

Figure S7. The effect of laser treatment on carbon paste coatings (electrodes). Cross-sectional SEM images comparing laser-treated and untreated (No Laser) regions of various carbon paste coatings. Panels (**a**) and (**b**) show Sun Chemical GST 4500 ink; (**c**) and (**d**) depict Gwent paste coating; and (**e**) and (**f**) display Henkel paste coating. The images illustrate that laser treatment has a significant effect on the surface roughness of the GST and Gwent pastes, visibly increasing roughness in the treated areas. In contrast, the Henkel coating remains relatively compact and unaffected in morphology after laser exposure.

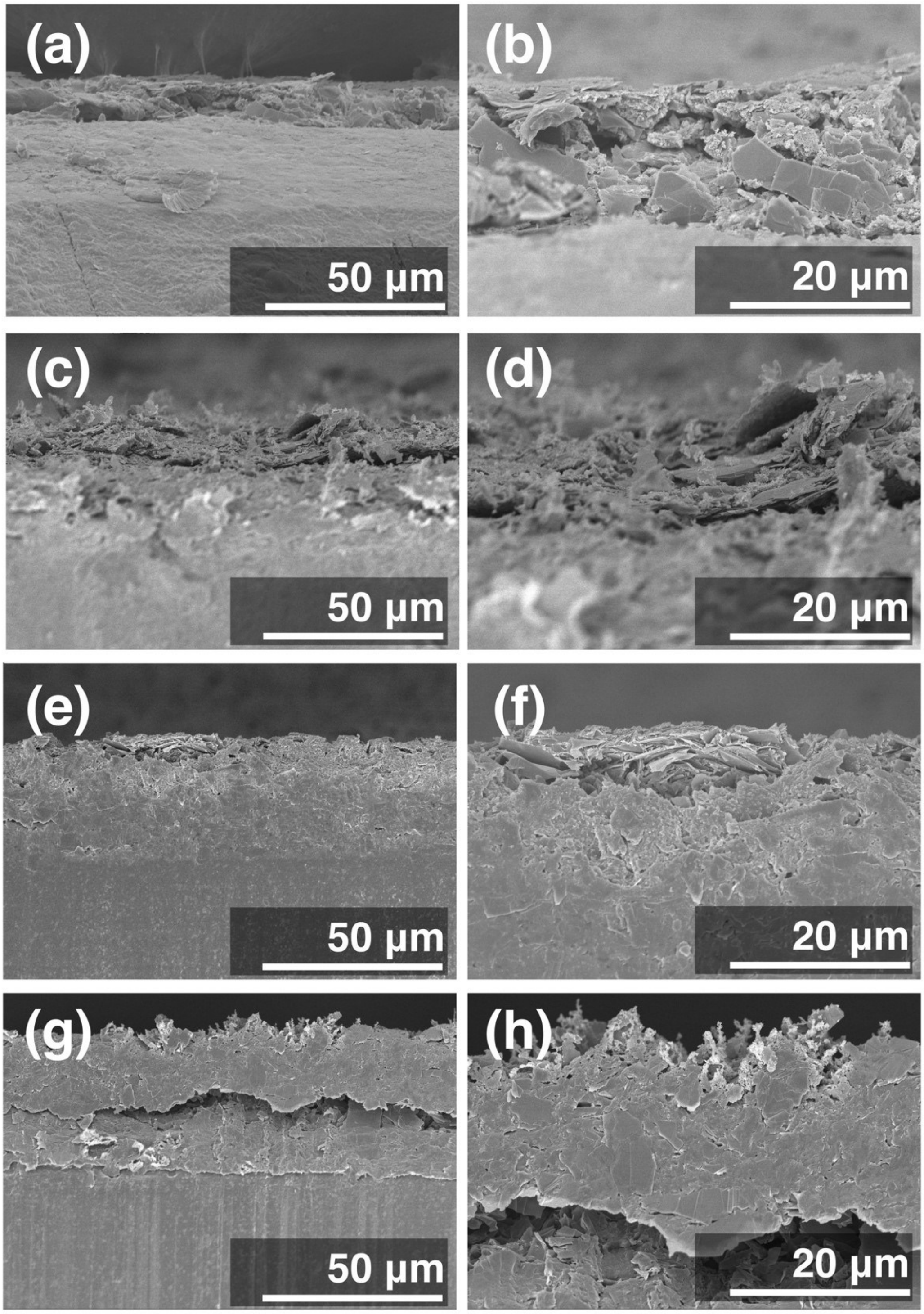

Figure S8. SEM cross-sectional images from electrodes printed using Gwent (**a-d**) and Henkel (**e-h**) carbon pastes. Images **a**, **b**, **e** and **f** correspond to as-printed electrodes. Images **c**, **d**, **g** and **h** correspond to laser-treated electrodes. Laser action roughens the surface as binder is ablated away. Dendrite-like structures appear on the graphite which are thought to be highly crystalline graphite responsible for the enhanced electrochemical response observed.

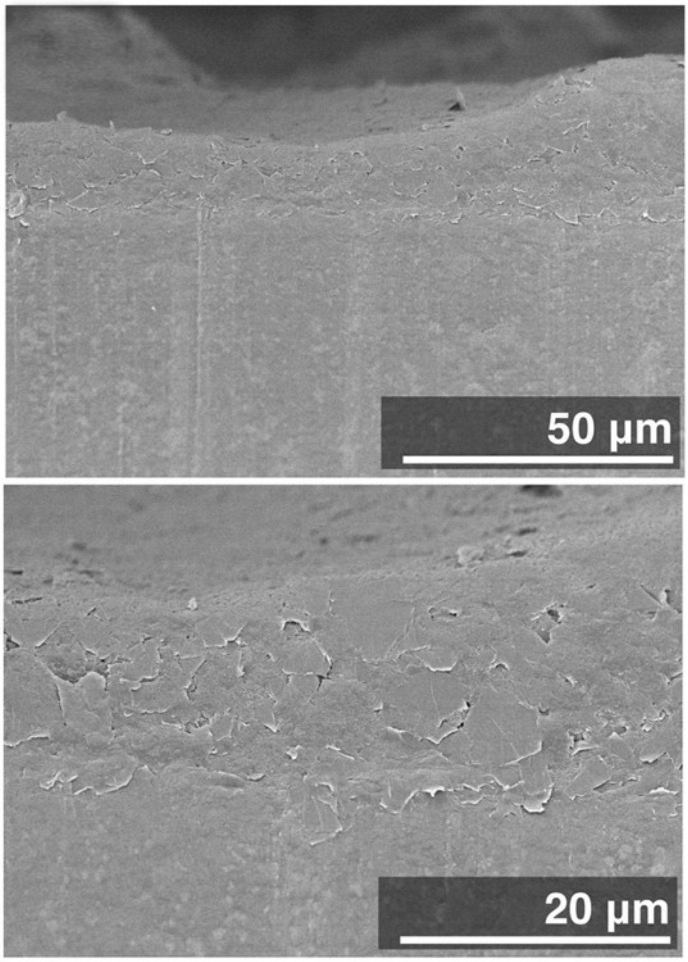

Figure S9. SEM cross-sectional images from a GST graphite screen printed electrode. The images show a compact structure with minor surface irregularities and a smooth surface compared to laser-ablated samples.

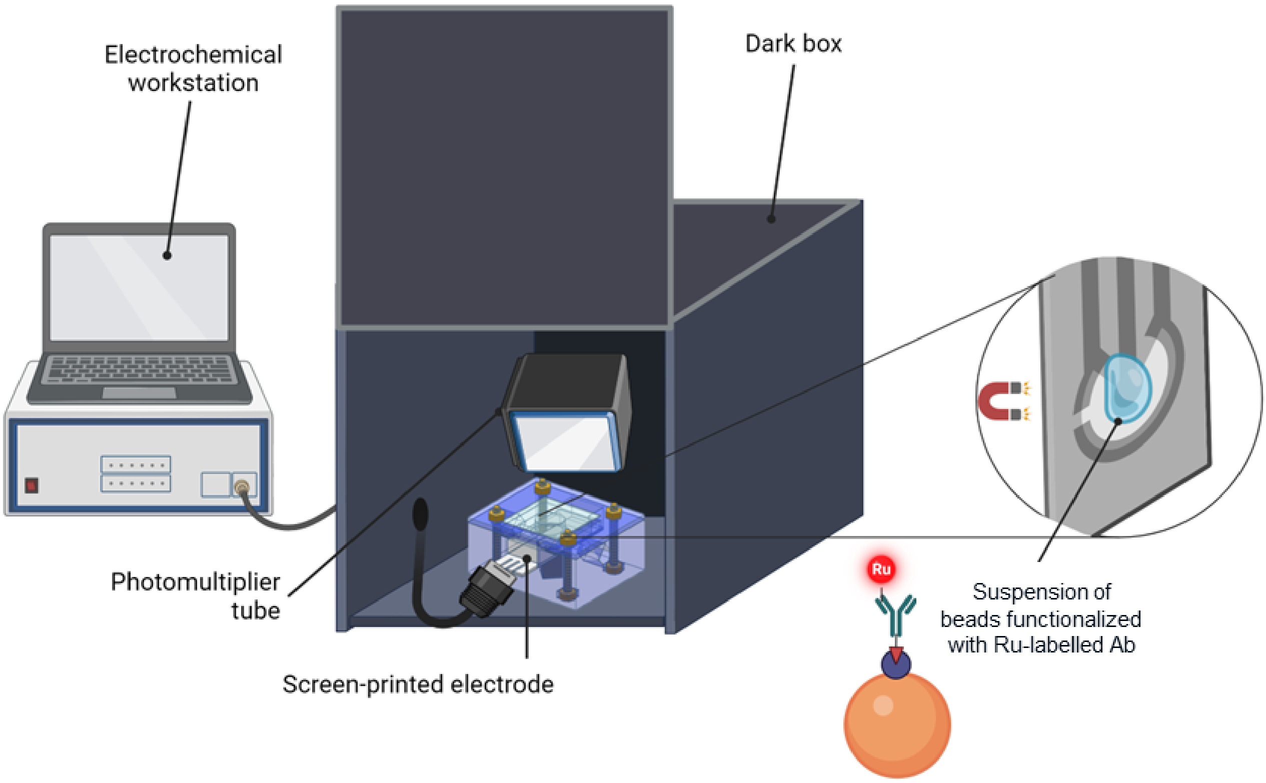

Figure S10. Schematic representation of quantitative ECL analysis of antibody-labeled magnetic microbeads. A photomultiplier tube (Hamamatsu R928) was positioned at a fixed distance above the 3D-printed electrochemical cell to detect ECL signals. To minimize external light interference, both the cell and the PMT were housed within a dark box. A high-voltage power supply with a trans-impedance amplifier (Hamamatsu C6271) provided a 750 V bias to the PMT, triggered externally by the potentiostat's DAC module. The amplified PMT output signal was acquired by the potentiostat's ADC module (BioLogic SP-300) to generate light/current/voltage graphs.

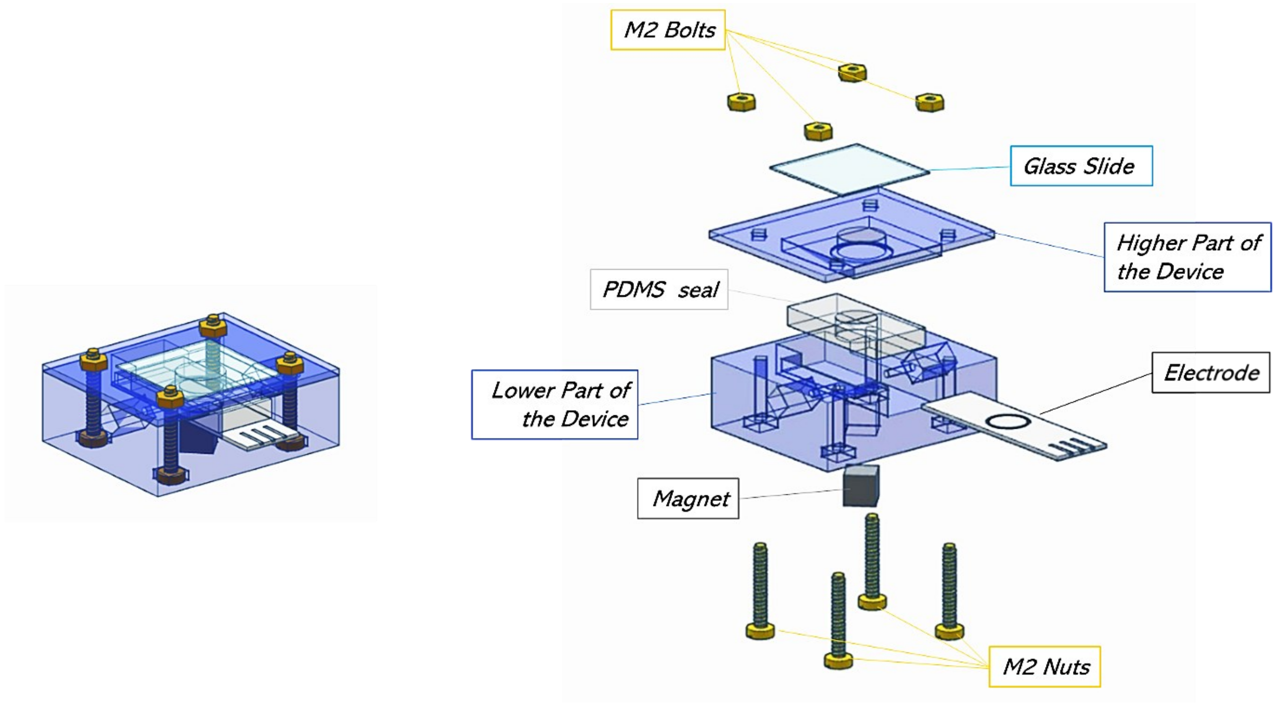

Figure S11. Schematic representation of homemade 3D printed cell. Each component is depicted with different colors: lower and higher parts of the device (blue), magnet underneath the electrode (gray), electrode (white), glass-slide (transparent light blue), o-ring (green), PDMS piece as a spacer (transparent gray), nuts and bolts (yellow). The custom cell was fabricated using a Fused Deposition Modeling (FDM) printer (Ultimaker S33D).

Supplementary Movie 1. Single-bead ECL intensity recording during the CV on lasered GST SPCE. **a)** ECL emission during the cyclic voltammetry obtained from the elaboration of the images obtained by ECL microscopy; **b)** multiple frames image as a function of the potential recorded during the ECL microscopy experiments. Scanning potential from 0 V to 2 V and reverse (vs. Ag/AgCl, 3 M KCl); acquisition time 0.2 s, scale bar image 10 µm.

References

(1) Alba, A. F.; Fernández-de Luis, R.; Totoricaguena-Gorriño, J.; Ruiz-Rubio, L.; Sánchez, J.; Vilas-Vilela, J. L.; Lanceros-Méndez, S.; del Campo, F. J. Understanding Electrogenerated Chemiluminescence at Graphite Screen-Printed Electrodes. *Journal of Electroanalytical Chemistry* **2022**, *914*, 116331. https://doi.org/10.1016/j.jelechem.2022.116331.
